# Encouraging efficacy of a comprehensive therapy consisting of sintilimab, bevacizumab biosimilar IBI305, hypo-fractionated intensity-modulated radiotherapy, and oxaliplatin for a maxillary metastasis from hepatocellular carcinoma: A case report and literature review

**DOI:** 10.3389/fonc.2022.941454

**Published:** 2022-11-23

**Authors:** Xuexia Liang, Qiaodan Liu, Wei Yao, Shuqin Zhu

**Affiliations:** ^1^ Department of Cancer Center, The Fifth Affiliated Hospital of Sun Yat-sen University, Zhuhai, China; ^2^ Guangdong Provincial Key Laboratory of Biomedical Imaging, Guangdong Provincial Engineering Research Center of Molecular Imaging, the Fifth Affiliated Hospital of Sun Yat-sen University, Zhuhai, China; ^3^ Department of Pathology, The Fifth Affiliated Hospital of Sun Yat-sen University, Zhuhai, China

**Keywords:** hepatocellular carcinoma, maxillary metastasis, PD-1 inhibitor, bevacizumab, chemoradiotherapy

## Abstract

Oro-maxillo-facial metastasis from hepatocellular carcinoma (HCC) is very rare, and reports on treating maxillary metastasis from HCC are unavailable. Anti-angiogenesis therapy combined with immunotherapy represented by programmed cell death 1 (PD-1) or its ligand (PD-L1) inhibitor has become the standard treatment of advanced HCC. However, integrating chemoradiotherapy into immunotherapy–bevacizumab combination therapy has not been reported. Here, we presented a Chinese woman with maxillary metastasis from HCC who achieved a nearly complete response (CR) to a quadruple treatment scheme consisting of a PD-1 monoclonal antibody (sintilimab), bevacizumab biosimilar IBI305, hypo-fractionated intensity-modulated radiotherapy (hfIMRT), and concurrent oxaliplatin. This comprehensive treatment is an innovative and effective therapy for advanced HCC.

## Introduction

Tumor metastasis to the oro-maxillo-facial region accounts only 1% of all malignant oral lesions. The most common metastasis to the oro-maxillo-facial region is from the primary tumor of the lungs, kidney, and breast, and the most often involved areas are the cervical lymph nodes, mandible, and gingiva (1, 2). Maxillary metastasis from hepatocellular carcinoma (HCC) is rare, and nearly 60% were found before primary HCC was diagnosed. Men were more frequently afflicted by this kind of metastasis (3). Treatment of maxillary metastasis from HCC was rarely reported.

HCC is a common cancer and the fourth leading cause of cancer-related death worldwide. Chronic hepatitis virus infection and consumption of aflatoxin-contaminated food are the most common etiological factors (4). Liver can be defined as an immunological organ because antigens from the gastrointestinal tract *via* the portal vein can be recognized and presented by various immune cells in the hepatic sinusoids. The priming immune cells interact with hepatocytes and hepatic stellate cells in the Disse space. Under pathological conditions, gut-derived antigens lead to chronic carcinogenic inflammation and an immune inhibitory microenvironment, which is characteristic of overexpression of immune checkpoint molecules (such as PD-1, PD-L1, CTLA4, and LAG3), cytokines and chemokines, and T-cell exhaust (5). Based on the strongly immunosuppressive tumor microenvironment (TME), immune checkpoint inhibitor (ICI) therapy becomes a rational choice for HCC. ICI monotherapy or ICI combined with angiogenesis inhibitor or locoregional therapy or chemotherapy has become a new promising treatment for HCC (5, 6). The IMbrave150 trial and ORIENT-32 trial established PD-1/PD-L1 inhibitors combined with bevacizumab or its biosimilar IBI305 as a first-line systemic treatment for advanced HCC (7, 8). External beam radiotherapy (EBRT) and chemotherapy synergized with ICI enhanced antitumor effects (9, 10). So far, there was no study integrating ICI, bevacizumab, EBRT, and concomitant chemotherapy for advanced HCC. Here, we firstly presented a Chinese woman with maxillary metastasis from HCC who responded dramatically to a PD-1 inhibitor (sintilimab) and bevacizumab biosimilar IBI305 combined with concurrent chemoradiotherapy with hfIMRT and oxaliplatin.

## Case description

A 72-year-old Chinese woman was found to have a 6.5 cm × 6.3 cm, rounded, boundary-clear, heterogeneously arterial enhanced (**Supplementary Figure 1A**), and delayed washout (**Supplementary Figure 1B**) mass lesion in liver S2/3 *via* a computed tomography (CT) scan of the abdomen in June 2019. There was central ischemic necrotic area in the mass. She had latent chronic hepatitis B virus (HBV) infection. Serologic tests of viral markers were as follows: HBs Ag, positive; HBe Ag, negative; HBV DNA, 1,290 IU/ml; and anti-hepatitis C virus, negative. Tenofovir alafenamide fumarate 25 mg every day was orally prescribed for her. The tumor marker alpha-fetoprotein (AFP), tested in June 2019, was 32,510.3 ng/ml. She was diagnosed with early-stage HCC (T1bN0M0, stage IB of the American Joint Committee on Cancer staging system; stage A of the Barcelona Clinic Liver Cancer staging system). Hepatic segmentectomy (S2/3) was performed on 18 June 2019. The tumor mass was completely resected, with a microscopic negative operation margin. Pathological examination of the specimen was as follows: HCC, grade 2–3 (moderately to poorly differentiated), no microvascular invasion, and negative margin. The immunohistochemical (IHC) analysis revealed the following (**Supplementary Figure 1C**): PD-L1 (−), CK19 (−), CK-p (weakly +), CK8 (weakly +), hepatocyte (focal +), Glypican-3 (+), Ki-67 (approximately 20% +), and positive CD34, indicating capillary-like hepatic sinusoid. AFP level tested in July 2019 dropped to normal levels (<400 ng/ml) after surgery.

**Figure 1:**
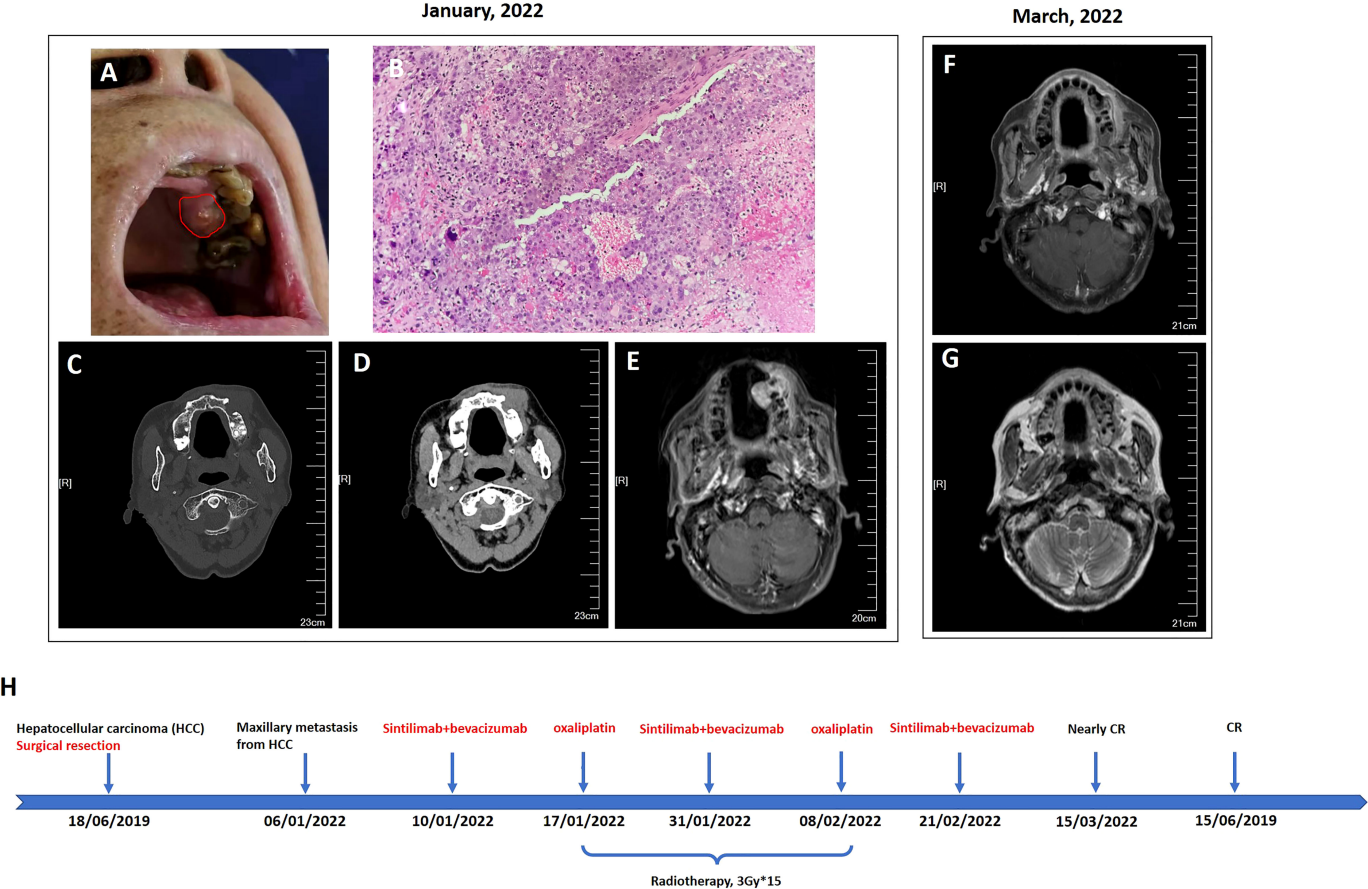
Oral examination revealed a mass on the upper left gingiva **(A)**, and head neck imaging indicated that it was an osteolytic lesion and formed a soft tissue mass protruding to the subcutaneous maxillo-facial region and oral cavity **(C–E)**; finally, it was diagnosed as maxillary metastasis from HCC after biopsy of the mass in the oral cavity (**B**, 200×). After comprehensive treatment of sintilimab plus bevacizumab biosimilar IBI305 plus radio-chemotherapy, the maxillary metastasis shrunk dramatically to nearly complete remission **(F, G)**. A time course of the entire case is shown in **(H)**.

**Figure 2:**
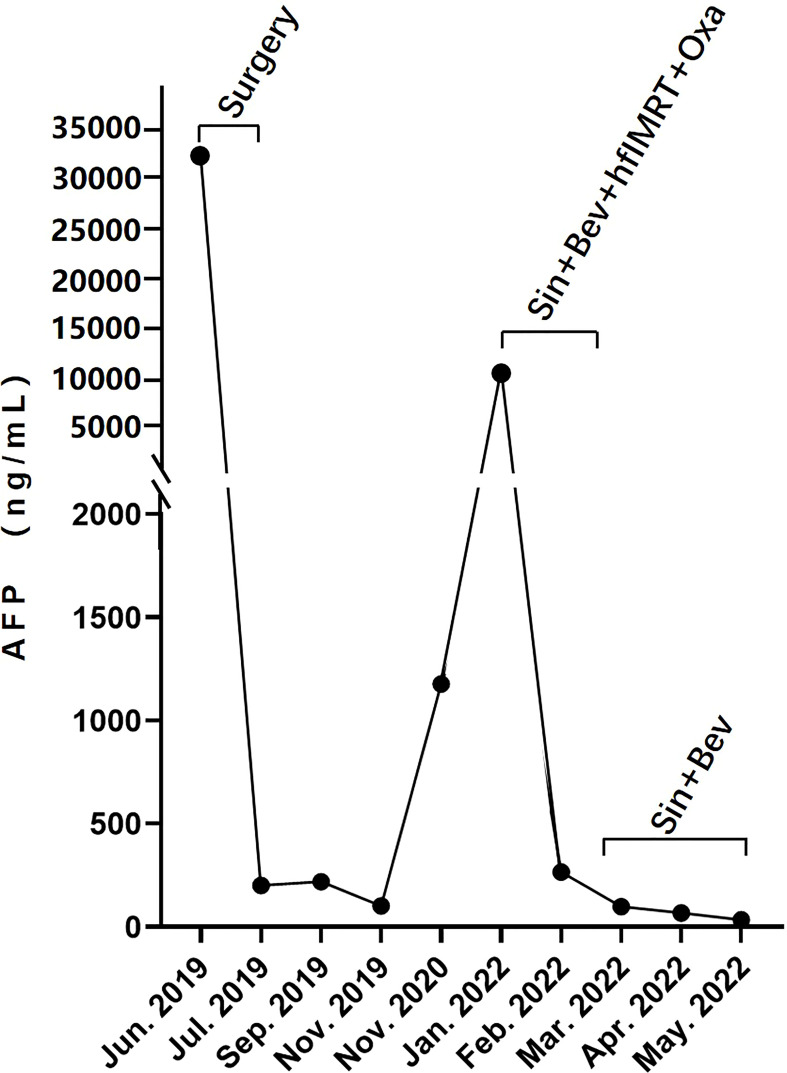
The dynamic changes of serous AFP level over time: AFP dropped to <400 ng/ml after surgical removal of an intrahepatic tumor in June 2019 and sintilimab-bevacizumab biosimilar IBI305–hfIMRT–oxaliplatin combination treatment in February 2022, and continued to decline gradually during the stage of maintenance treatment with sintilimab and bevacizumab biosimilar IBI305.

Serologic tumor biomarker test again revealed a high AFP level of 1,177 ng/ml on 20 November 2020. However, no tumor recurrence in liver or extrahepatic metastatic lesion was detected on CT of the chest and abdomen. After a year, the patient visited a dental clinic on 6 January 2022, complaining about a progressively enlarged, painful, bleeding, and hard soft tissue mass on the top left of the gingiva (**Figure 1A**). CT and MRI of the head and neck showed osteolytic destruction of left maxilla and a 2.8 cm × 2.2 cm enlarged soft tissue mass (**Figures 1C–E**). Pathological biopsy indicated poorly differentiated adenocarcinoma (**Figure 1B**), and results of IHC analysis were as follows (**Supplementary Figure 2**): CK-p (+), CK8/18 (+), hepatocyte (focal +), vimentin (−), p63 (−), CK5/6 (−), CK-H (−), CK7 (−), Ki-67 (approximately 70% +), and PD-L1 (tumor proportion score < 1% +). Her AFP test was 10,231.21 ng/ml. Considering imaging, biopsy, high AFP level, IHC analysis, and previous HCC history, she was diagnosed with maxillary metastases from HCC. Laboratory tests revealed a white blood cell count of 7.66 × 10^9^/L, with 70.4% neutrophils, a platelet count of 302 × 10^9^/L, and a prothrombin time [international normalized ratio (INR)] of 1.18. Her blood chemistry profile was as follows: total protein, 74.9 g/L; albumin, 40.0 g/L; total bilirubin, 9.9 μmol/L; aspartate aminotransferase (AST), 49.1 U/L; alanine aminotransferase (ALT), 23.1 U/L; alkaline phosphatase (ALP), 78 U/L; and gamma-glutamyl transferase, 25 U/L. She refused to take the genetic test, which was not covered by governmental resident medical insurance, because of low personal income. She also refused surgical resection and asked for noninvasive treatment whose reimbursement rate of medical insurance was more than 70%. Finally, with her informed consent and her meeting the requirements we delivered 3Gy×15 fractions by IMRT to the maxillary metastasis lesion concurrent with intravenously infused oxaliplatin (140 mg, every 21 days) 1 week after intravenous infusion of sintilimab (a PD-1 antibody, 200 mg, every 21 days) plus bevacizumab biosimilar IBI305 (600 mg, every 21 days). After hfIMRT, two cycles of oxaliplatin and three cycles of sintilimab and bevacizumab biosimilar IBI305, AFP level dropped to <400 ng/ml. On 15 March 2022, MRI showed that the maxillary metastasis had obviously shrunk and a nearly complete response (CR) was achieved according to the Response Evaluation Criteria in Solid Tumor version 1.1 (**Figures 1F, G**). A time course of the entire case is shown in **Figure 1H**. The changes in AFP are illustrated in **Figure 2**. The patient tolerated the combination treatment with slight adverse effects. There was no treatment modification, interruption, or discontinuation. A grade 1 increase in AST (73.2 U/L) and ALT (42.7 U/L), grade 1 anemia, grade 1 nausea, and grade 2 oral mucositis occurred. She continued maintenance treatment with sintilimab (200 mg, every 21 days) and bevacizumab biosimilar IBI305 (600 mg, every 21 days) until May 2022, and AFP level gradually declined (**Figure 2**).

## Discussion

HCC is the fourth most common cancer and the third most common cause of cancer-related death in China in 2015 (11). HCC extrahepatic metastasis usually involves the lungs and regional lymph nodes (12), and metastasis to the oro-maxillo-facial area is rare, which represents less than 1% of all oral neoplasms, with mandible more often involved than maxilla (1–3). This female patient had no lung metastasis, and the possible pathway of maxillary metastasis from HCC is through hematogenous dissemination *via* the portal vein–plexus venosus vertebralis–azygos vein connection (3). She had symptoms of oral pain and bleeding, and physical signs of a progressively enlarged gingival mass, which also showed similar imaging features to primary oral cancer. Therefore, diagnosing HCC maxilla metastasis should finally depend on pathological biopsy, IHC results, and tumor markers.

Targeting TME to enhance anticancer therapy is currently a treatment strategy of great interest. TME consists of tumor cells and their surrounding components, which are characterized by immune cells, vasculature, stroma cells, and extracellular matrix, and interact with each other. Distorted and leaky tumor vasculature leads to a TME characterized by hypoxia and increased interstitial fluid pressure (13). Hypoxia promotes the formation of immunosuppressive microenvironment *via* compromising the function of natural killer T cells, M1-type tumor-associated macrophages (TAMs), mature dendritic cells (DCs), and TH1 cells, and recruiting immunosuppressive regulatory T cells (Tregs), myeloid-derived suppressor cells (MDSCs), and M2-type TAMs (14, 15). Angiogenesis factors, including vascular endothelial growth factor (VEGF) and its receptors (VEGFRs), repress antigen-presenting cells (APCs) and potentiate the function of Tregs, MDSCs, and M2-type TAMs (13). Immunosuppressing cells secrete cytokines or chemokines, which promote angiogenesis, like interleukin-10 (IL-10) and matrix metallopeptidase (MMP)-9 from immature DCs or MDSCs or M2-type TAMs, IL-4, IL-5, and IL-13 from Tregs (16). Therefore, angiogenesis and immunosuppressive TME develop a reinforcing feedback loop to promote tumor progression. Anti-angiogenesis therapy can enhance the infiltration of CD8+ cytotoxic T cells into tumor tissue and the transportation efficiency of anticancer drugs *via* normalizing tumor vasculature and reducing hypoxia (17, 18). Decreased hypoxia *via* anti-angiogenesis drugs also promotes macrophage polarization to the M1 type and increases the secretion of angiogenesis-inhibiting molecules, such as interferon-γ from CD8+ T cells and CXCL9, CXCL10, and CCL21 from M1-type TAMs (16, 18). The anti-angiogenesis therapy and immunotherapy combination form a positive feedback loop, and this might be the reason for this combination therapy’s success in providing survival benefits. Based on the above mechanism and encouraging results of the IMbrave150 trial (7) and ORIENT-32 trial (8), we chose anti-angiogenesis therapy combined with ICI as the basic systemic treatment for this patient. Sintilimab, a recombinant humanized anti-PD-1 monoclonal antibody, has been shown to have excellent and durable antitumor activity against various malignancies, including HCC (19). Bevacizumab biosimilar IBI305, a recombinant anti-VEGF humanized monoclonal antibody, exhibits similar pharmacokinetic profile, efficacy, and safety compared to Avastin (20, 21). Therefore, finally, we treated this patient with equivalent and inexpensive sintilimab and bevacizumab biosimilar IBI305 with the expectation of a robust and durable therapeutic effect.

EBRT is recommended for HCC treatment by the National Comprehensive Cancer Network (NCCN) guideline in various scenarios (www.nccn.org) and is an immune response modifier for immune-oncology. On the one hand, irradiation of a tumor leads to immunogenic cell death (ICD), which will result in the release of danger-associated molecular patterns (DAMPs), and activates cGAS/STING/IFN-I signaling, which promotes the production and release of cytokines and chemokines (22). IFN-I and DAMPs (such as ATP and HMGB-1) can stimulate the maturation of DCs and promote the anticancer function of cytotoxic T lymphocytes (CTLs) and natural killer cells (NKs) (23). On the other hand, irradiation induces an immunosuppressive TME *via* activating the ROS/hypoxia-inducible factor 1 alpha (HIF-1α) signaling pathway, which results in the overexpression of genes [such as CXCL12, transformation growth factor-β (TGF-β), and VEGF] that promote immunosuppression *via* recruiting Tregs and MDSCs and M2 polarization of TAMs (23). Oxaliplatin-based chemotherapy (FOLFOX regimen) is recommended as a first-line systemic treatment in certain circumstances by the NCCN guideline for HCC. Similarly, platinum-based chemotherapy also has a dual-directional modifying effect on tumor immunity. On the one hand, platinum-based medication such as oxaliplatin exhibits an immune-stimulatory effect through increasing tumor cell expression of human leukocyte antigen-1 (HLA-1), enhancing the antigen presentation capacity of tumor cells, and alleviating immune evasion in cancer. Platinum-based chemotherapy induces ICD, which increases the availability of DAMPs within tumor tissue and tumor-associated antigens to APCs, and augments anticancer T-cell response. On the other hand, chemotherapy promotes synthesis of local cytokine and chemokine including immunostimulating and immunosuppressive molecules. Chemotherapy can increase tumor expression of PD-L1 and facilitate immune escape (24). Concomitant chemoradiotherapy had a synergistic anticancer effect and achieved an objective response rate of 46.8%–48%, an improved median overall survival (OS) of 9.8–13.5 months, and a median progression-free survival (PFS) of 6.9–7.8 months in patients with locally advanced HCC (25–27). Thus, in order to control the maxillary metastasis from HCC as early as possible here, we chose chemoradiotherapy consisting of IMRT-designed hypo-fractionated EBRT with 3Gy*15f delivered to tumor and concurrent oxaliplatin (85 mg/m^2^, igtt, q3w). The tumor biological equivalent dose was 58.5 Gy (tumor α/β = 10). This chemoradiotherapy scheme was a mild treatment combination for this 72-year-old female patient without ≥grade 3 adverse events. To counteract the immunosuppressive effect of chemoradiotherapy, sintilimab and bevacizumab biosimilar IBI305 were infused intravenously 1 week before radio-chemotherapy with the hypothesis that the sintilimab–bevacizumab combination medication normalized tumor vasculature and preheated immune microenvironment in advance, and would be maintained until tumor progression.

Combining EBRT with ICI immunotherapy and anti-angiogenesis therapy has caught the attention of clinical researchers. In Sahebjam et al.’s prospective research, adding hypo-fractionated EBRT (6Gy*5) to pembrolizumab (200 mg, every 3 weeks) plus bevacizumab (10 mg/kg, every 2 weeks) achieved an overall response rate (ORR) of 78% and a median OS of 9.3–13.5 months in PD-L1-negative recurrent high-grade glioma (28). Regarding HCC, a retrospective study showed an ORR of 40% and a disease control rate of 86.7% in advanced HCC receiving palliative EBRT combined with ICIs and anti-angiogenic molecular treatment. The median PFS and OS were 4.6 months and 21 months, respectively (9), and there are several ongoing prospective trials investigating EBRT plus ICIs plus anti-angiogenesis treatment for advanced HCC (ChiCTR1900027102, NCT05010434, NCT05096715, NCT04857684, NCT05137899, and ChiCTR2200056068). However, there is no study reporting the comprehensive treatment of ICI plus anti-angiogenic therapy plus EBRT plus oxaliplatin for HCC so far.

This is the first report to describe an old Chinese woman with rare HCC maxillary metastasis receiving sintilimab–bevacizumab biosimilar–hfIMRT–oxaliplatin combination treatment despite negative tumorous PD-L1 expression and achieving nearly CR. PD-L1 expression-based treatment decision-making and response prediction may be insufficient. A study reported that frequencies of microsatellite instability high (MSI-H) status and high tumor mutational burden (TMB-H) were very low in hepatobiliary cancer (29). More prognostic biomarkers are highly necessary. This patient refused next-generation sequencing gene testing; thus, which genetic characteristics contributed to her treatment success were unclear, which was a major limitation of this case report. It is possible that the rapid reduction of tumor burden of single maxillary metastasis by early-initiated chemoradiotherapy enhanced the anticancer effect of the sintilimab–IBI305 combination, and this may explain the treatment success here.

The encouraging response of a case with maxillary metastasis from HCC treated by a quadruple therapy demonstrated that the sintilimab–bevacizumab–EBRT–oxaliplatin combination was an innovative and efficacious treatment strategy. The dosing and sequencing of radio-chemotherapy in an ICI–bevacizumab setting need further study. A diagnosis of maxillary metastasis from HCC should be confirmed by pathological biopsy. Tumor PD-L1 expression, among other things, should be investigated to predict the response in this combination treatment setting.

## Data availability statement

The original contributions presented in the study are included in the article/**Supplementary Material**. Further inquiries can be directed to the corresponding author.

## Ethics statement

Ethical review and approval were not required for this study on human participants in accordance with the local legislation and institutional requirements. The patient provided their written informed consent to participate in this study. Written informed consent was obtained from the patient for the publication of any potentially identifiable images or data included in this article.

## Author contributions

XL carried out this study, and collected data with QL, WY and SZ. XL drafted the manuscript. All authors contributed to the article and approved the submitted version.

## Funding

This work was supported in part by a grant from the Scientific-Technologic Foundation of Zhuhai City, China (Grant No. 20191210E030088) and a grant from Guangdong Province Medical Science and Technology Research Fund Project (Grant No. A2021295).

## Acknowledgments

We appreciate the teamwork of all authors and thank the patient and her family.

## Conflict of interest

The authors declare that the research was conducted in the absence of any commercial or financial relationships that could be construed as a potential conflict of interest.

## Publisher’s note

All claims expressed in this article are solely those of the authors and do not necessarily represent those of their affiliated organizations, or those of the publisher, the editors and the reviewers. Any product that may be evaluated in this article, or claim that may be made by its manufacturer, is not guaranteed or endorsed by the publisher.

## Supplementary material

Supplementary Figure 1 Abdominal CT scanning revealed a rounded, boundary-clear, heterogenous arterial enhanced **(A)** and delayed wash out mass lesion in liver S2/3 with central necrotic area **(B)** in Jun 2019. This mass was diagnosed as HCC by hematoxylin-eosin (HE) **(C)** and immunohistochemistry staining of paraffin-embedded tumor tissue after hepatic segmentectomy with negative surgical margin.

Supplementary Figure 2 Oral mass on the upper left gingiva was finally diagnosed as maxillary metastasis from HCC by hematoxylin-eosin and immunohistochemistry staining after biopsy.
