# Supplementary figures and images for: Encouraging efficacy of a comprehensive therapy consisting of sintilimab, bevacizumab biosimilar IBI305, hypo-fractionated intensity-modulated radiotherapy, and oxaliplatin for a maxillary metastasis from hepatocellular carcinoma: A case report and literature review
Source: Front Oncol. 2022 Nov 23;12:941454. doi: 10.3389/fonc.2022.941454 (PMC9727184; doi:10.3389/fonc.2022.941454)

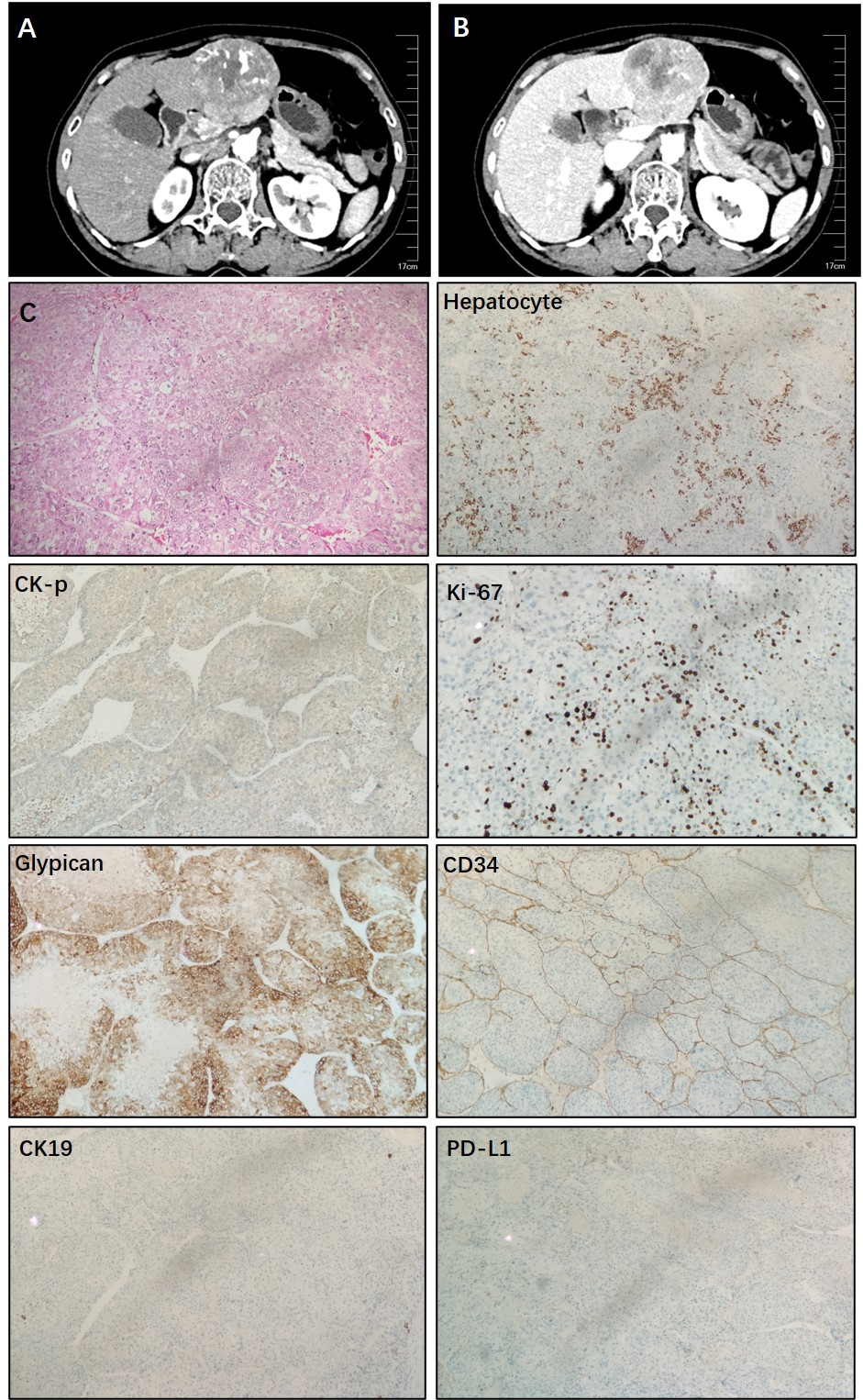

Supplement: Supplementary Figure 1 — Abdominal CT scanning revealed a rounded, boundary-clear, heterogenous arterial enhanced (A) and delayed wash out mass lesion in liver S2/3 with central necrotic area (B) in Jun 2019. This mass was diagnosed as HCC by hematoxylin-eosin (HE) (C) and immunohistochemistry staining of paraffin-embedded tumor tissue after hepatic segmentectomy with negative surgical margin. [file Image_1.jpeg]

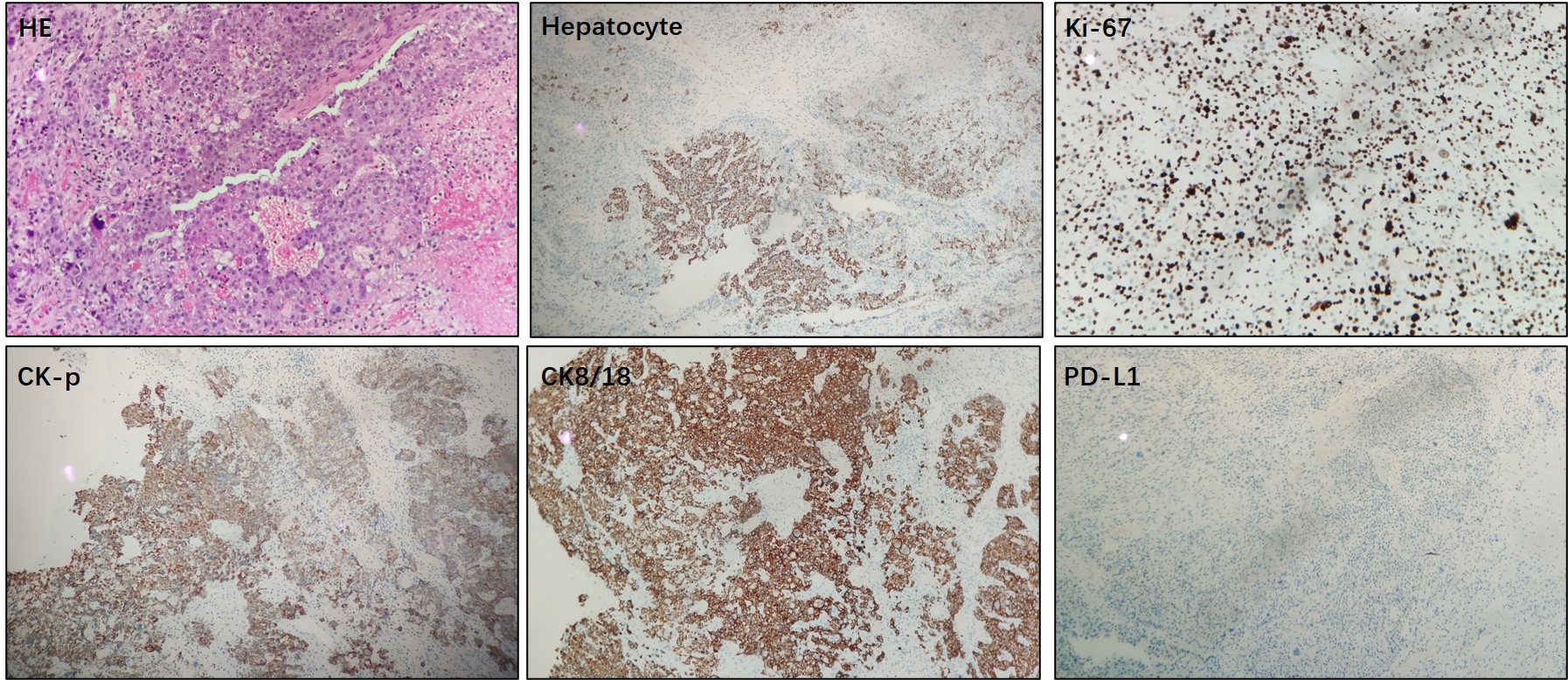

Supplement: Supplementary Figure 2 — Oral mass on the upper left gingiva was finally diagnosed as maxillary metastasis from HCC by hematoxylin-eosin and immunohistochemistry staining after biopsy. [file Image_2.jpeg]
